# Barriers and Enablers of Service Access and Utilization for Children and Adolescents With Attention Deficit Hyperactivity Disorder: A Systematic Review

**DOI:** 10.1177/10870547231214002

**Published:** 2023-12-11

**Authors:** Kaitlyn McKenna, Sithara Wanni Arachchige Dona, Lisa Gold, Angela Dew, Ha N. D. Le

**Affiliations:** 1 Deakin University, Burwood, Australia

**Keywords:** ADHD, children, adolescent ADHD, service use, review

## Abstract

**Objective:**

To update a systematic review of the literature on the barriers and enablers of service access and utilization for children and adolescents with a diagnosis, or symptoms of attention deficit/hyperactivity disorder (ADHD), from the perspective of caregivers, clinicians, and teachers.

**Methods:**

Five databases were searched for peer-reviewed literature published from May 2012 to March 2023. Two independent reviewers completed a two-stage screening process and quality assessment.

**Results:**

Of 4,523 search results, 30 studies were included. Five main themes were generated: 1) Awareness of ADHD, 2) Stigma, 3) Parental choice and partnerships, 4) Education services as an integral component, 5) Referrals, waiting times, and logistics. More than half of the studies reported poor acknowledgement, expertise of ADHD, and stigma.

**Conclusion:**

Findings highlight the need for ongoing ADHD education for all involved and policy changes to service delivery systems to increase the availability of health providers with specialist ADHD expertise.

## Introduction

### Background

Attention-deficit/hyperactivity disorder (ADHD) is a common neurodevelopmental condition among children (i.e., ≤18 years old), with a global prevalence of 3 to 7% of children and adolescents (Polanczyk et al., 2007, 2015; Sayal et al., 2018; Thomas et al., 2015). ADHD in children is characterized by inattention or hyperactivity, which interferes with the child’s functioning, development, and ability to perform daily activities such as academic participation (Reynolds & Kamphaus, 2013). ADHD may also be associated with other conditions, such as specific learning difficulties, developmental coordination disorder, oppositional defiant disorder, mood, and anxiety disorders. These comorbidities can complicate the process of early diagnosis for children experiencing symptoms (Fredriksen et al., 2014; Harpin, 2005; Young et al., 2021).

The negative health and social impacts of ADHD have been well established (Fredriksen et al., 2014; Hamed et al., 2015; Harpin, 2005; Peasgood et al., 2016; Sikirica et al., 2015). Unmanaged ADHD can have significant effects on the quality of life of children and their families (Danckaerts et al., 2010; Harpin, 2005). Compared to their peers without ADHD (Harpin, 2005), children with ADHD may experience reduced ability to concentrate, poor social skills, poor academic outcomes, and suicidal thoughts (Ljung et al., 2014). The impacts of ADHD in childhood can flow into adolescence and young adulthood, with experiences of employment dismissal, antisocial and criminal behavior, teenage pregnancy, and substance abuse (Harpin, 2005).

Early diagnosis and treatment of ADHD in children is important because it can lead to a better quality of life, positive outcomes in daily activities, and improved academic performance (Hamed et al., 2015; Mattingly et al., 2017; Sikirica et al., 2015). The process of an ADHD diagnosis differs between countries globally; many countries across Europe and Australia have a “gatekeeper” system whereby a referral from a general practitioner or primary care clinician provides access to specialist ADHD diagnosis and treatment services (Sayal et al., 2018; Tatlow-Golden et al., 2016; Young et al., 2021). In the United States, the health system is delivered privately, and ADHD diagnosis and management is typically provided by primary care physicians/pediatricians or psychiatrists (Hamed et al., 2015; Sayal et al., 2018).

There are various approaches to ADHD treatment and management including pharmacotherapy, psychoeducation, and cognitive behavioral therapy (Kooij et al., 2019). The initiation of comprehensive multimodal treatment can reduce negative experiences and increase children’s quality of life, with ongoing monitoring of treatment outcomes (Harpin, 2005; Sikirica et al., 2015). Research on the uptake and utilization of ADHD treatment services demonstrates that treatment uptake is inconsistent with the rates recommended for ADHD management and outlines high unmet medical and social needs in children with ADHD (Le et al., 2023), particularly for families with a lower socio-economic status (Sevastidis et al., 2023). Differences in healthcare service and treatment utilization can be attributed to the socio-economic status of families and national service delivery models (Sevastidis et al., 2023). Nevertheless, families who receive adequate treatment services for their child’s ADHD can be faced with significant healthcare and treatment costs (Gupte-Singh et al., 2017; Le et al., 2023; Sciberras et al., 2017).

The literature has contributed to increased awareness of ADHD, and improvements have occurred in service provision for ADHD diagnosis and treatment in children over the last few decades (Young et al., 2021). However, unmet medical and social needs for children and adolescents with ADHD mean symptoms continue, and caregivers can face a challenging journey to obtain diagnosis and treatment for their child (Fridman et al., 2017; Park et al., 2020; Sikirica et al., 2015; Young et al., 2021). There are gaps between the rates of children diagnosed with ADHD and those receiving treatment for their symptoms (Danielson et al., 2018). For example, the United States National Survey of Children’s Health 2016 revealed that of the 8.4% of children diagnosed with ADHD in the United States, nearly a quarter (23%) of these children were not receiving either medication or behavioral therapy treatment for their condition (Danielson et al., 2018).

Similarly, the ADHD-Europe Survey 2020 identified long waiting lists for children and families accessing ADHD diagnostic services (Stivala, 2021). This finding did not align with the early intervention recommendation for ADHD in the National Institute for Health and Clinical Excellence guidelines implemented in the United Kingdom and referred to across Europe (Stivala, 2021). The survey findings also described geographical disparities in accessing ADHD specialist services due to inadequate national funding (Stivala, 2021). Also, in Europe, the Caregiver Perspective on Pediatric ADHD (CAPPA) survey found that almost a third of families experienced a high level of difficulty in obtaining a diagnosis for their child. More than half of the CAPPA survey respondents believed that insufficient resources were available, identifying gaps in access to diagnosis and supportive care services (Fridman et al., 2017).

Identifying the barriers and enablers of service access and utilization in service planning and provision is important to improve the experience and ease of accessing ADHD diagnosis and treatment for families with symptomatic/diagnosed children. Equitable access to services and ongoing service utilization will improve the quality of life for children and families managing ADHD (Hamed et al., 2015; Sikirica et al., 2015; Young et al., 2021).

Caregivers can face several barriers throughout the process of ADHD diagnosis and treatment for their child, such as structural and logistical challenges (Baweja et al., 2021; Young et al., 2021). Access to appropriate ADHD services varies significantly between regions and countries, and variations in referral pathways to specialists and waiting times to access assessment can make it time-consuming and difficult for caregivers to navigate access to supportive care for ADHD management in children (Fridman et al., 2017; Young et al., 2021). Lack of recognition and support from educational settings and clinicians can extend the time to accessing diagnosis and receiving treatment (Sikirica et al., 2015). Challenges such as the availability of services, financial, limited insurance coverage, and transportation barriers for people living in regional areas can also influence access to specialist services (Fridman et al., 2017; Sikirica et al., 2015; Spencer et al., 2021; Wright et al., 2015).

For some families, there may be a stigma associated with a child’s behavior or an ADHD diagnosis whereby caregivers may be reluctant to pursue diagnosis and treatment, and health providers may be hesitant to assess and diagnose a child with ADHD because of social stigma (French et al., 2019; Wright et al., 2015; Young et al., 2021). These barriers can make it challenging to navigate health and education systems and receive timely access to diagnosis, treatment and support that aligns with national guidelines and recommendations.

A systematic review by Wright et al. (2015) found that the barriers and facilitators to care pathways occurred across different levels, from individual, organizational to societal, and may be influenced by decision-making, and knowledge and attitudes of parents, teachers, and professionals. The review highlighted the influence of parental decision-making in accessing services, the detrimental impact of stigma and labeling on families, their willingness to engage in care, and the desirability of school-based interventions for effective treatment and care for children and adolescents. Wright et al. (2015) synthesized literature only up to April 2012, specifically on the access to services for children and adolescents at-risk of ADHD.

Diagnostic criteria and treatment guidelines have evolved over the past decade (Australian ADHD Guideline Development Group, 2022; Coghill et al., 2023) and current shortfalls in ADHD diagnosis rates and service delivery systems have been recently identified (Coghill et al., 2023; Sayal et al., 2018; Young et al., 2021), which may have impacted upon families accessing and utilizing services for their child with ADHD. Therefore, an update on the barriers and enablers of service access and utilization, specifically for children and adolescents ≤18 years old, is needed. The current systematic review updates and extends the synthesis of the literature on the barriers and enablers to service access and utilization for children and adolescents (≤18 years old) with a diagnosis or symptoms of ADHD by exploring the recent literature on this topic from May 2012 to March 2023. These barriers and enablers were explored from the perspective of parents, clinicians, and teachers. For the current review, the term “service” refers to the services for the diagnosis, treatment, and management of ADHD in children and adolescents, which include: 1) clinical services, such as primary care clinicians, pediatric services, psychiatrists, and mental health providers, and 2) educational services, such as school personnel, including school-based mental health professionals.

## Methodology

This systematic review was reported based on the PRISMA guidelines 2020 (Page et al., 2021) and was registered with the International Prospective Register of Systematic Reviews (PROSPERO: CRD42023418207) in April 2023 (McKenna et al., 2023).

### Search Strategy

The search strategy was developed by the primary reviewer (KM) through consultation with the review team. The primary reviewer conducted the search on the following databases via EBSCOhost: MEDLINE Complete, APA PsycInfo, CINAHL Complete, ERIC, and Global Health. The searches were limited to articles written in English, peer-reviewed, and published from 01 May 2012 to 19 March 2023 to capture contemporary literature. The search involved keywords (Appendix A, Table A1) and subject headings (Appendix A, Table A2), for the four main concepts of the search strategy: 1) Child/adolescent, 2) ADHD, 3) service access/utilization, and 4) barrier/enabler.

### Inclusion and Exclusion Criteria

For studies to be included, the criteria were set as: (1) peer-reviewed publications written in English and published from 01 May 2012 onward, (2) studies with participants of children and adolescents ≤18 years old (at least 50% of participants either clinically diagnosed and/or symptoms of ADHD), parents, carers, teachers of children with ADHD, and/or health professionals, and (3) studies exploring the barriers and enablers of ADHD service access and utilization. Studies were excluded if they did not meet the above criteria or if they were literature/systematic reviews, case studies, opinion pieces, editorials, and abstracts.

### Study Selection Process and Data Extraction

Results from the database search were exported to Endnote (The EndNote Team, 2020) and then imported to Covidence Systematic Review Software (Covidence, 2020). Two independent reviewers (KM and SWAD) completed a two-stage screening process: 1) title and abstract screening, and 2) full-text screening. Any disagreements were resolved through discussion with the senior reviewer (HL).

A data extraction template (Table 1) was developed in Covidence Systematic Review Software. The primary reviewer (KM) completed the data extraction, which was cross-checked by the second reviewer (SWAD). The extracted information described the author, focus, country, participants, setting, study design, key findings, and theme of each included study.

**Table 1.** Study characteristics.

Author, (year)	Focus	Country	Sample characteristics	ADHD status	Study period	Setting	Study design	Key findings	Themes
Araujo et al. (2017)	To explore the emotional, social, and cultural experiences of Latino youth with ADHD symptoms and their families	United States	13 Latino caregivers of children (aged 7–10 years) with ADHD symptoms	ADHD symptoms in child	2014–2015	Collaborative Life Skills program in three U.S. schools	Qualitative research	Three major themes: acculturation, family dynamics, and language. Racism, and language difficulties negatively affected caregiver’s ability to seek and receive care for their child with ADHD. Ethnicity and language barriers affected school and health institution access	1. Clinician, teacher, and parental acknowledgment and expertise/knowledge of ADHD 2. Stigma
Barry et al. (2016)	To describe the process and descriptive outcomes of initiating school-based screening procedures	United States	1,044 children identified as at-risk for a diagnosis of ADHD based on teacher screening, (average of 8.68 years old), 636 parents of the screened children	Vanderbilt AD/HD Diagnostic Teacher Rating Scale (VADTRS)	Unclear	Five school districts located in the central and southern regions of the state of Mississippi	Cross sectional study	Lack of parental knowledge regarding ADHD characteristics may act as a barrier to diagnosis through school screening. Reluctance to seek treatment once parent is informed their child is at-risk due to denial, blame, uncertain about medication, and lack of resources (time, finance)	4. Education services as an integral component 5. Referrals, waiting times and logistics
Coletti et al. (2012)	To describe results from formative research activities to obtain stakeholder input for a physician-delivered intervention targeting adherence to stimulant regimens	United States	27 parents of children (aged 5–12 years) with an ADHD diagnosis (average of 9.35 years old)	Diagnosis of ADHD by a child psychiatrist in the outpatient clinic	2009	Ambulatory care child psychiatry clinic of a large north-eastern suburban teaching hospital	Qualitative research	Barriers to medication treatment included fears of personality changes and medication side effects, and social norms of medication as “overused.” Enablers to treatment include shared decision making and collaborative care with medical providers. Attitudes to psychiatric treatment and the impact of social and emotional influences affected a parent’s decision to initiate medication treatment for their child with ADHD	1. Clinician, teacher, and parental acknowledgment and expertise/knowledge of ADHD 2. Stigma 3. Importance of parental choice and partnerships
Graves (2017)	Explored caregivers help-seeking experience in negotiating barriers in their environment to accessing mental health treatment for their children	United States	11 mothers with at least one child receiving mental health treatment and attended three or more sessions with a mental health provider	Confirmed ADHD diagnosis in child	Unclear	Public housing authority in an urban area	Qualitative research	Four categories in a central theme of filters of influence: recognition of concerning behaviors, the decision to seek formal mental health treatment, the decision to accept mental health treatment, and the decision to remain in mental health treatment. Mothers were ambivalent about medication treatment due to cultural beliefs and side effects and expressed concerns with mental health professionals as unresponsive to the children’s needs	2. Stigma 4. Education services as an integral component
Hooven et al. (2018)	To understand barriers to compliance with primary care office contacts for ADHD medication management	United States	306 children (aged 6–12 years) with an ADHD-related diagnosis	ADHD-related ICD-9/ICD-10 diagnosis	June 2015 to May 2016	Three general pediatrics practices at a single academic medical center	Other: exploratory and retrospective chart review	Parent work schedule and the child’s school and activity schedule were reported as barriers to attending an ADHD medication visit. Increased distance was the main structural barrier identified in the chart review, and high caregiver strain was reported in interviews, suggested as an influencing factor to service utilization	5. Referrals, waiting times and logistics
Johansson et al. (2023)	To understand patient characteristics that predict engagement barriers during behavior therapy for ADHD	United States	121 adolescents (aged 11–16 years) with ADHD and parents who received evidence-based behavior therapy for ADHD	DSM Criteria, structured parent interview, and parent/teacher symptom and impairment ratings	Unclear	Supporting Teens Autonomy Daily (STAND) evidence-based treatment for adolescents with ADHD at a university clinic	Cross sectional study	Comorbidity of ODD, and parental ADHD predicted barriers to engagement in adolescent behavioral therapy for ADHD	5. Referrals, waiting times and logistics
Moody (2017)	Identify the community, political, and social barriers to diagnosing ADHD among Black children	United States	15 parents, three teachers, an administrator, and a counselor	Children with disciplinary/learning issues	March 2015	A school in a low-income, predominantly Black community in Memphis, Tennessee	Qualitative research	Several barriers to diagnosis were identified which included parents’ attitudes, lacking an understanding of ADHD, and teachers misinformed about ADHD, refusing to make referrals for children due to labeling and contributing to the stigma (cultural)	1. Clinician, teacher, and parental acknowledgment and expertise/knowledge of ADHD 2. Stigma
O’Dell et al. (2022)	Perspectives of PCCs within a large, rural health system on factors affecting pediatric ADHD care	United States	25 Primary Care Clinicians treating pediatric ADHD	None	June 2018 to October 2018	Rural health system in the Northeast United States	Qualitative research	Mental health stigma and limited access to psychosocial treatments for ADHD were barriers to care access, behavioral health providers as an enabler to increasing access to psychosocial treatment. Coordination between clinicians, families and the school were an enabler to treatment utilization. PCCs have taken on behavioral health access gaps and geographic/systemic challenges to engaging families and schools in care due to increased responsibility for pediatric ADHD care	1. Clinician, teacher, and parental acknowledgment and expertise/knowledge of ADHD 2. Stigma 4. Education services as an integral component
Paidipati et al. (2022)	To qualitatively describe the major contextual influences that impact family management for ethnically diverse children with ADHD	United States	50 caregivers of children (aged 5–12 years) with ADHD	Confirmed diagnosis of ADHD in child	2016–2017	Large pediatric academic institution located in a north-eastern city in the United States	Mixed-methods study	Barriers included inadequate access to services: extended wait times for mental health services, high turnover of staff, inconsistent care and communication, transportation. ADHD as a stigmatized diagnosis, experience racial bias, and concerns around medication. Finance (unaffordable services), insurance (services refusing insurance), and policy issues impacted access and utilization of services for ADHD management. Educational providers facilitated resources and guidance for access to services. There are major contextual barriers and facilitators of care management	1. Clinician, teacher, and parental acknowledgment and expertise/knowledge of ADHD 2. Stigma 3. Importance of parental choice and partnerships 4. Education services as an integral component 5. Referrals, waiting times and logistics
Pastor et al. (2017)	To examine the use of medication and other nonmedication mental health services	United States	Parent reports of children (aged 4–17 years) with ADHD	Confirmed diagnosis of ADHD in child	2010–2013	National Health Interview Survey	Cross sectional study	Differences in insurance status, and the type of insurance were associated with differences in use/non-use of treatment services and health professionals. Health and school service utilization, medication use and first prescriber as a psychiatrist/psychologist was more common in publicly insured children than privately and uninsured children. Uninsured children were less likely to receive mental health care and special education	5. Referrals, waiting times and logistics
Saulsberry et al. (2020)	To use a strengths-based approach to determine African American parents’ skills and strategies for management of children with ADHD	United States	16 African American parents of children (aged 4–17 years) with ADHD	Child diagnosed with ADHD by a psychologist, psychiatrist, or other doctor	Unclear	Outpatient pediatrics clinic of a large urban Midwestern medical center, an outpatient child psychiatry clinic associated with the medical center, and federally qualified health centers	Qualitative research	Positive relationship with a physician was an enabler for service utilization. African American parents experienced barriers and challenges such as stigma (racism), less/poorer quality of care, less availability of services, labeling instead of a formal diagnosis, and the child’s symptoms not being recognized and acknowledged	1. Clinician, teacher, and parental acknowledgment and expertise/knowledge of ADHD 2. Stigma 3. Importance of parental choice and partnerships 4. Education services as an integral component
Shippen et al. (2022)	To understand communities’ experiences with ADHD across development and to explore the barriers/facilitators to adequate services	United States	6 adolescents with ADHD, 5 caregivers, 6 teachers, 5 school mental health providers	Confirmed ADHD diagnosis in child	Unclear	Two public urban high schools in a mid-Atlantic school district where approximately 78.6% of students are Black	Qualitative research	Two main themes: 1) developmental changes affecting the presentation of ADHD among Black high school students, 2) contextual factors influencing Black adolescents with ADHD. Contextual factors involved individual, familial, school-based, and community/systems-level factors. School based barriers to care involved lack of staff training and insight regarding ADHD, contributing to the stigma. A systematic barrier was a lack of access to mental health services outside of school, and health insurance coverage (difficulty obtaining, to accessing services). School-based services were an enabler to care access	1. Clinician, teacher, and parental acknowledgment and expertise/knowledge of ADHD 2. Stigma 4. Education services as an integral component
Spencer et al. (2021)	To examine the process by which families engage in ADHD treatment and to identify targets for an intervention to improve engagement in care	United States	41 caregivers of youth (aged 3–17 years) in treatment of ADHD	Confirmed diagnosis of ADHD in child	June 2018 to October 2019	Pediatric clinics at Boston Medical Centre	Qualitative research	Six stages of engagement from recognizing the first symptoms in the child, to preparing for the child’s future for when they are independent. Parental hesitation, and fear of stigma caused reluctance to seek a diagnosis. Barriers to accessing healthcare providers included not being acknowledged, and logistics such as cost and transport. Trusting relationships with, and validation from providers enabled ongoing service utilization for treatment. Stages were considered as milestones, and there were barriers to overcome each stage	1. Clinician, teacher, and parental acknowledgment and expertise/knowledge of ADHD 2. Stigma 3. Importance of parental choice and partnerships 5. Referrals, waiting times and logistics
Zima et al. (2013)	To examine whether parent perceptions about care vary among children who drop out of or stay in publicly funded care for (ADHD) and to explore whether parent perceptions are predictive of staying in care over time	United States	546 caregivers of children (aged 5–12 years) receiving ADHD care	Receiving care defined as at least one visit with a primary diagnosis of ADHD, or at least 1 claim for a stimulant prescription	November 2004 to September 2006	One of the largest managed-care Medicaid programs in primary or specialty mental healthcare outpatient settings	Cohort study	Intangible and logistical barriers were reported by parents such as speaking with their doctor, stigma, lack of information, available time to get help, finance, and transportation. Common misconceptions and poor ADHD knowledge were evident. Fewer barriers did not improve the likelihood of staying in care	5. Referrals, waiting times and logistics
Lu et al. (2022)	To explore parents’ perspectives on living with a child with ADHD and their perspectives on treatment options for their child with ADHD	United States and Canada	23 parents of children (aged 6–12 years) with ADHD	Confirmed ADHD diagnosis in child	January 2019 to March 2020	Sub-study of the Micronutrients for ADHD in Youth (MADDY) study	Qualitative research	Two main themes: impact of ADHD on families within and outside the home and enabling appropriate and accessible treatments for families. Finding the right fit for child’s needs with health professionals was a key facilitator to successful ADHD treatment. Major barriers to treatment access included limited options and lack of choice, financial barriers, and limited insurance coverage to accessing holistic treatment plans. Patient/family-centered, step-wise approaches essential to provide optimal care to children with ADHD and their families	3. Importance of parental choice and partnerships 5. Referrals, waiting times and logistics
Benevides et al. (2017)	To identify predisposing, enabling, and need characteristics associated with lack of access to therapy, and frequently occurring reasons for access problems.	Canada	Caregivers of children with ADHD (2005–2006: 10,511 children), (2009–2010: 10,055 children)	Confirmed ADHD diagnosis in child	Data sets: 2005–2006, 2009–2010	National Survey of Children with Special Health Care data sets	Cross sectional study	Lack of school resources and high financial costs as a reason for poor therapy access and unmet therapy need	5. Referrals, waiting times and logistics
Fridman et al. (2017)	To report caregivers’ experiences of ADHD diagnosis, behavioral therapy (BT), and supportive care for children/adolescents with ADHD	Canada	3,616 caregivers of a child (aged 6–17 years) with an ADHD diagnosis and receiving pharmacotherapy at least 6 months prior to the study	Confirmed ADHD diagnosis in child	November 2012 to April 2013	Caregiver Perspective on Pediatric ADHD survey	Cross sectional study	Wide variation across ten European countries regarding the availability and quality of ADHD services. Caregivers reported a great deal of difficulty in obtaining a referral and a formal ADHD diagnosis. Caregivers were dissatisfied with support/assistance from schools. Local diagnostic pathways, or specific model of health care can act as a barrier or enabler to timely diagnosis (time from noticing symptoms to a formal ADHD diagnosis)	5. Referrals, waiting times and logistics
Ogundele et al. (2022)	Explored the Covid-19 pandemic experiences of United Kingdom-based Pediatricians who assess and treat children and young people with ADHD	United Kingdom	62 Pediatricians who provide ADHD services for children and young people	None	March 2020 to June 2020	Delegates who attended the George-Still Forum annual scientific meeting in 2019 (UK-wide ADHD Clinical Network)	Cross sectional study	COVID19 pandemic presented various challenges to the assessment, diagnosis and treatment of children and young people with ADHD. School closure resulted in lack of assessment information and pediatricians ceased new ADHD assessments. Pediatricians’ were cautious around prescribing medications and dosages. Telemedicine was an important enabler for continued service access utilization for existing patients not requiring physical assessment	4. Education services as an integral component
Rezel-Potts et al. (2021)	Exploring perspectives on barriers to accessing general practice or specialist services for their child/young person for ADHD and broader health reasons and strategies for improvement	United Kingdom	8 parents of children/young people with ADHD	Confirmed ADHD diagnosis in child	February 2019	Members of a support group for parents/carers of children and young people with ADHD	Qualitative research	Communication with health professionals and the need to self-advocate to overcome barriers to their child’s ADHD being recognized and acknowledged. Barriers to accessing healthcare services included difficulty getting an appointment, conflicting times with school, inconvenient locations, and the child’s condition not being severe enough	1. Clinician, teacher, and parental acknowledgment and expertise/knowledge of ADHD 3. Importance of parental choice and partnerships 5. Referrals, waiting times and logistics
Sayal et al. (2015)	To investigate predictors of and barriers to specialist health service use for mental health or behavioral problems amongst at-risk children whose schools participated in randomized controlled trial	United Kingdom	83 parents of baseline-high-scoring children	High scoring children were defined as children who had 6 or more symptoms relating to one of the DSM-IV ADHD subtypes in the previous study	Unclear	5-year follow-up study of children who participated in a cluster RCT regarding school interventions (20 Local Education Authority areas) for children at risk of ADHD	Other: Longitudinal follow-up	The main barriers reported by parents were a lack of information about who could help, professionals did not listen when asking for help, wait times, a perception that no one could help, and poor communication with health professionals. Other barriers included trying to find the right services, cost, and availability of services. Parents had requested and received referrals from primary healthcare, and education professionals	1. Clinician, teacher, and parental acknowledgment and expertise/knowledge of ADHD 4. Education services as an integral component 5. Referrals, waiting times and logistics
Flood et al. (2019)	To explore the experiences of a selection of Irish parents of children who have been prescribed medicines for ADHD	Ireland	10 English-speaking adults who had direct experience of making decisions relating to treating their child with medication for ADHD (nine mothers and one father)	Confirmed ADHD diagnosis in child	May 2016 to October 2016	Members of ADHD Ireland Family Support Group, and clinical settings	Qualitative research	4 categories: (1) Influences of initial challenges arising from having a child with ADHD (challenges in accessing services) (2) Struggling to make a risk/benefit decision (struggle to get a referral from primary care) (3) The impact of initial experiences with medication, and (4) Regaining some control over management and decision-making. The urgency around the need for treatment and having poor ADHD knowledge was evident. Delays in accessing services, and variable primary care experiences added to the sense of crisis at the time of diagnosis	5. Referrals, waiting times and logistics
Laugesen et al. (2017)	To explore parental experiences of how healthcare practices and healthcare professionals in hospital clinics in Denmark influence everyday life of the parent with a child with ADHD	Denmark	15 parents of children (aged 5–12 years old) diagnosed with ADHD	Confirmed ADHD diagnosis in child	February 2015 to November 2015	Two general pediatric outpatient clinics and two child and adolescent mental health clinics	Qualitative research	Three main themes: when the house of cards collapses in everyday life, treading water before and after receiving the ADHD diagnosis, and healthcare as a significant lifeline. Importance of healthcare services and professionals being accessible and treating the families with respect. Allies in healthcare and trusting relationships were found to be enablers of providing support for families	1. Clinician, teacher, and parental acknowledgment and expertise/knowledge of ADHD 3. Importance of parental choice and partnerships
Laugesen et al. (2020)	To explore and describe everyday life and hospital-based healthcare experiences and utilization in families of children with ADHD in Denmark	Denmark	15 parents of children (aged 5–12 years) with a diagnosis of ADHD. 11,360 children were identified to have been given a diagnosis of ADHD in hospitals	Current diagnosis of ADHD, and ICD-10codes F90.0. F90.1, and F98.8 were used to identify children with ADHD	2014–2017	2 general pediatric outpatient clinics, 2 child and adolescent mental health clinics in public hospitals. Danish nationwide registers of children born between 1995 and 2002	Mixed-methods study	Three mixed methods findings: 1) Long-term prospects of having concerns recognized, 2) Family stressors influencing everyday life and healthcare, and 3) The importance of healthcare in navigating the persistent challenges of everyday life. Use of medical and psychiatric services in hospitals for children with ADHD higher in first 12 years of life, than children without ADHD. Highlighted importance of families being recognized and accepted in in hospital-based healthcare services from early childhood	1. Clinician, teacher, and parental acknowledgment and expertise/knowledge of ADHD 5. Referrals, waiting times and logistics
Lien et al. (2015)	Examined the extent to which characteristics of family and health care providers predict treatment initiation, mode, and termination among preschool children with newly diagnosed ADHD	Taiwan	A cohort of 3,583 pre-schoolers with ADHD (initial ADHD diagnosis between ages of 3 and 5 years old)	Confirmed ADHD diagnosis in child	Unclear	2001–2007 data from the National Health Insurance Research Database in Taiwan	Other: Retrospective longitudinal design	Diagnosis from a psychiatrist as a possible enabler for treatment initiation of combined treatments (multimodal). Utilization of treatment services may be influenced by limited availability of pediatric care in primary care settings. Family and service provider characteristics showed to have differential effects on initiation, mode, and termination of treatment	5. Referrals, waiting times and logistics
Tzang et al. (2014)	Explores barriers to seeking help for children with attention deficit hyperactivity disorder (ADHD) in Taiwan	Taiwan	Caregivers of 104 children with ADHD	Diagnostic and Statistical Manual, Fourth Edition criteria: Confirmed ADHD diagnosis in child	Unclear	Outpatient department of Mackay Memorial Hospital in Taipei	Cross sectional study	The misleading idea of practicing sensory integration training, and parental attitudes of medication treatment have been identified as possible barriers to refusing medical treatment, and not seeking advice from a pediatric psychiatrist for their child’s treatment	5. Referrals, waiting times and logistics
Yamauchi et al. (2015)	To examine the factors associated with a time lag between initial parental concern about ADHD symptoms and the first visit to a hospital in Japan that offers child psychiatric services	Japan	4,323 caregivers, sample of 387 children (less than 19 years old) with ADHD	Confirmed ADHD diagnosis in child	September 2008 to March 2009	16 leading hospitals that specialize in child psychiatric services	Cross sectional study	Caregivers experienced challenges in knowing which services to consult regarding mental health problems in their children (lack of knowledge about where to seek help), which contributed to the time length between first noticing symptoms and visiting a child psychiatric service. Mothers’ education level could be an enabler to early assessment and diagnosis of ADHD	1. Clinician, teacher, and parental acknowledgment and expertise/knowledge of ADHD 5. Referrals, waiting times and logistics
Anand et al. (2018)	To provide a quantitative description of the factors affecting the help-seeking pathway	India	50 children (aged 5–15 years), and parents of those children	Screened for ADHD using Conners’ Parent Rating Scale-Revised: Short Form and behavioral observation, Diagnostic and Statistical Manual of Mental Disorders fourth edition and the Kiddie Schedule for Affective Disorders and Schizophrenia Present and lifetime version	March 2010 to March 2011	Pediatrics Outpatient Department: tertiary care multispecialty hospital at New Delhi, India	Cross sectional study	Families of urban geographical location, and higher maternal education level sought earlier consultation with services. Lack of awareness of treatment options and associated stigma contributed to a delay in seeking treatment. The majority of children were referred by school teachers. Teachers and GPs formed an important source of referral. Parents’ help‑seeking behavior was affected by various sociocultural beliefs	1. Clinician, teacher, and parental acknowledgment and expertise/knowledge of ADHD 2. Stigma 4. Education services as an integral component 5. Referrals, waiting times and logistics
Safavi et al. (2016)	To investigate the prevalence of ADHD among elementary school students of Shahrekord and to assess the obstacles preventing patients from accessing mental health services	Iran	109 students (aged 6–12 years) were identified with ADHD	Child Symptom Inventory-4 (CSI-4) presenting six or more symptoms of ADHD were categorized as suspected ADHD	2012–2013	Elementary schools in Shahrekord	Cross sectional study	Obstacles to accessing mental health services were categorized into health and mental system, disease stigma, lack of need, financial, and negative expectations. The barriers to treatment were influenced by beliefs of patients and their families, while others were the result of limitations in the health care system	1. Clinician, teacher, and parental acknowledgment and expertise/knowledge of ADHD 2. Stigma 5. Referrals, waiting times and logistics
Al-Mohsin et al. (2020)	To assess the knowledge, experiences, and attitudes of mothers with ADHD children toward ADHD and identify their common sources of information and service barriers from the perspectives of these mothers in the Dammam, AI-Khobar, and Al-Qatif areas	Saudi Arabia	132 mothers of ADHD children (<9 years old)	Confirmed ADHD diagnosis in child	November 2018 to January 2019	Private and government sector special education schools and day care centers in Al Khobar, Dammam, and Qatif cities of Saudi Arabia	Cross sectional study	Explored source of information, reason for visiting services, and treatment delays. Identified barriers related to 1) health care services, 2) mothers/caregivers, 3) non-human resources and finance. Highlighted important role of clinicians in assessing mother’s knowledge and correct misconceptions, and importance of available information in enabling parents informed decision-making	1. Clinician, teacher, and parental acknowledgment and expertise/knowledge of ADHD 5. Referrals, waiting times and logistics
Honorio Neto et al. (2021)	To investigate neuro/community pediatricians and child and adolescent psychiatrists’ attitudes and practice regarding ADHD in private and public services in Brazil	Brazil	32 neuro/community pediatricians and 65 child and adolescent psychiatrists	None	Unclear	Brazilian neuro/community pediatricians and child and adolescent psychiatrists	Mixed-methods study	Treatment barriers identified by participants were a lack of shared care for ADHD cases. School observation was helpful for early identification of children with ADHD, however communication between health services and schools was poor, and support was only offered in specific circumstances. Challenges to diagnosis include time and cost to diagnosis and treat, assessments from different professionals. Lack of understanding of ADHD in parents and teachers negatively impacted assessment and treatment	1. Clinician, teacher, and parental acknowledgment and expertise/knowledge of ADHD 2. Stigma 4. Education services as an integral component

### Quality Assessment

Included studies were assessed for methodological quality by two independent reviewers (KM and SWAD) using the Mixed Methods Appraisal Tool (2018 version) (MMAT) developed by Hong et al. (2018) for appraising the quality of empirical studies (Appendix B, Table B1). Disagreements between the two reviewers were resolved through discussion. The tool included five categories of study designs: 1. Qualitative, 2. Quantitative randomized controlled trials, 3. Quantitative non-randomized, 4. Quantitative descriptive, and 5. Mixed methods, with five methodological quality criteria questions for each category. The scoring technique involved a numerical and categorical score whereby each question was rated as yes (1), no (0), or cannot tell (0.5), and overall quality was rated as low (1–2), medium (3), or high (4–5) based on the total score (Evangelio et al., 2022; Hong et al., 2018).

### Analysis

Common themes for the narrative synthesis were generated by following the process of thematic analysis outlined by Braun and Clarke (2006).

The primary researcher read all the included studies and selected relevant data excerpts from qualitative and quantitative articles. Using an inductive approach, the researcher completed the data coding with semantic codes (i.e., codes are descriptive and remain close to the original data that is written/stated), and some latent codes (i.e., interpretive and seeks meaning beyond what is written/stated in the data). Themes were generated from combined codes based on patterned meaning and frequency.

## Results

### Study Characteristics

**Figure 1.**
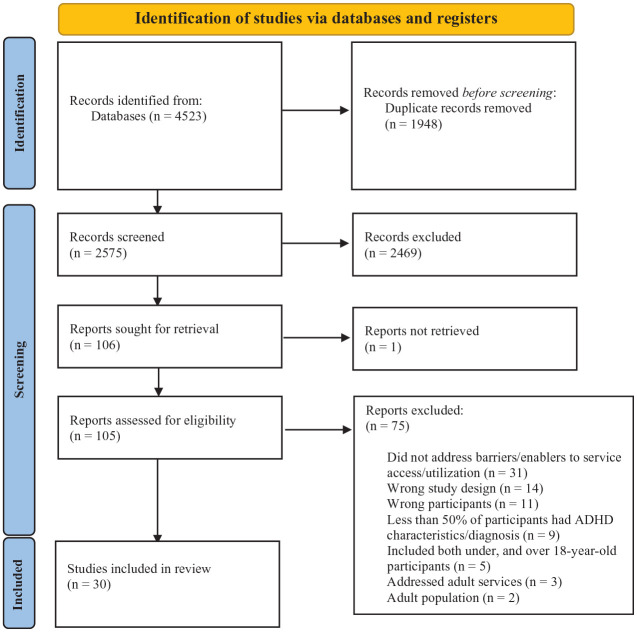
PRISMA diagram.

The search yielded 4,523 studies, of which 30 were included in the final analysis (Figure 1). There was an inter-rater agreement of 81% among the two reviewers for full-text screening. The quality of 96% of studies (*n* = 29) were rated as high, the remaining study (*n* = 1) was rated as medium (Appendix B, Table B1).

Included studies represented a wide range of countries across four continents; seventeen from North America (United States [*n* = 14], Canada [*n* = 2] and United States and Canada [*n* = 1]), six from Europe (United Kingdom [*n* = 3], Denmark [*n* = 2], Ireland [*n* = 1]), six from Asia (Taiwan [*n* = 2], Japan [*n* = 1], India [*n* = 1], Iran [*n* = 1], Saudi Arabia [*n* = 1]) and one from South America (Brazil [*n* = 1]).

Of the 30 included studies, 13 used a quantitative descriptive study design such as cross-sectional surveys or analysis of existing data sets from medical records and national surveys (Al-Mohsin et al., 2020; Anand et al., 2018; Barry et al., 2016; Benevides et al., 2017; Fridman et al., 2017; Hooven et al., 2018; Johansson et al., 2023; Lien et al., 2015; Ogundele et al., 2022; Pastor et al., 2017; Safavi et al., 2016; Tzang et al., 2014; Yamauchi et al., 2015). Two studies used a quantitative longitudinal design (Sayal et al., 2015; Zima et al., 2013); there were no randomized design studies. Thirteen studies used a qualitative design that included focus groups and interviews, analyzed through grounded theory, ethnography, and phenomenological approaches (Araujo et al., 2017; Coletti et al., 2012; Flood et al., 2019; Graves, 2017; Laugesen et al., 2017; Lu et al., 2022; Moody, 2017; O’Dell et al., 2022; Paidipati et al., 2022; Rezel-Potts et al., 2021; Saulsberry et al., 2020; Shippen et al., 2022; Spencer et al., 2021). Two studies used a mixed method design consisting of both quantitative and qualitative methods (Honorio Neto et al., 2021; Laugesen et al., 2020) (Table 1). The majority of the participants in the included studies were caregivers with children diagnosed with or experiencing symptoms of ADHD (*n* = 24); other studies reported data from health professionals, including pediatricians, psychiatrists, and primary care clinicians (*n* = 3), a combination of caregivers, teachers, and health professionals associated with ADHD diagnosis/treatment (*n* = 2), and an analysis of a population data set (*n* = 1). In terms of ADHD diagnosis, 21 studies involved parent-reported ADHD diagnosis of their child, and five studies conducted ADHD screening or diagnosis of children as part of the sampling process; the remaining four studies did not report ADHD diagnosis in children as the participants were health professionals/teachers.

### Barriers and Enablers

Heterogeneity in quantitative studies prevented a quantitative meta-analysis, therefore, all study designs were included in the narrative synthesis. Relevant quantitative data and statistics were identified and incorporated into the development of the themes.

**Figure C1.**
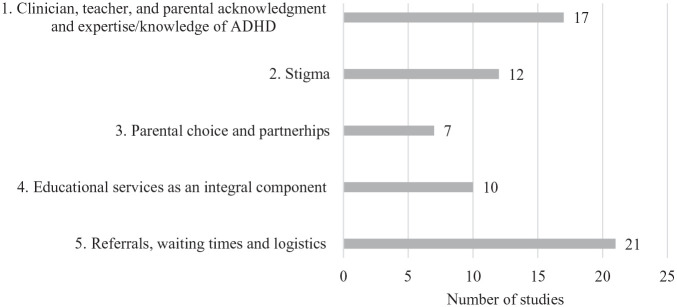
Barriers and enablers for ADHD service access and utilization.

Five main themes were generated from the included studies: (1) Clinician, teacher, and parental acknowledgment and expertise/knowledge of ADHD (*n* = 17), (2) Stigma (*n* = 12), (3) Parental choice and partnerships (*n* = 7), (4) Education services as an integral component (*n* = 10), and (5) Referrals, waiting time, and logistics (*n* = 21) (Appendix C, Figure C1).

#### Clinician, Teacher, and Parental Acknowledgment and Expertise/Knowledge of ADHD

A major theme generated from the included studies was the influence of clinician and parental awareness, acknowledgment, and expertise of ADHD on enabling access to referral, diagnosis, and treatment for children in need. More than half of the included studies (*n* = 17) reported poor acknowledgment of ADHD symptoms among clinicians (*n* = 5) and teachers (*n* = 6) as a barrier to accessing services, along with a lack of acknowledgment and awareness of ADHD from caregivers (*n* = 10), and a lack of expertise among clinicians (*n* = 2).

##### Lack of Acknowledgment From Clinicians and Teachers

Caregivers consistently reported a lack of acknowledgment from clinicians and teachers when seeking assessment or treatment for their child. Reported barriers included clinicians who were not ready to acknowledge the issue or act on behalf of the child, ignored and dismissed caregivers’ concerns, or questioned the legitimacy of the child’s ADHD diagnosis or treatment plan (Rezel-Potts et al., 2021; Sayal et al., 2015; Spencer et al., 2021). For example, in a 5-year follow-up study, 54% of caregivers who were service users, and 6% of non-service users reported that professionals did not listen when asking for help. Caregivers expressed feeling frustrated after being dismissed by health professionals who were unsupportive or lacked understanding of ADHD and struggled to have their concerns about their child recognized (Laugesen et al., 2017, 2020). Other caregivers experienced challenges when healthcare professionals questioned the legitimacy of the child’s ADHD diagnosis or treatment plan, causing parents to feel judged as bad parents (Rezel-Potts et al., 2021).

In addition to the clinical setting, caregivers also experienced a lack of acknowledgment from education providers and teachers, often reported by caregivers as associated with their Latino (Araujo et al., 2017), and African American ethnicity (Saulsberry et al., 2020). Both caregivers and teachers reported how a lack of teacher understanding and insight of ADHD in children can result in reduced opportunities and availability of support for children in need (Coletti et al., 2012; Moody, 2017; Paidipati et al., 2022; Shippen et al., 2022).

##### Lack of Awareness/Acknowledgment of ADHD Among Caregivers

Caregivers’ own poor awareness and acknowledgment of ADHD was also reported as a reason for delay in help-seeking or accessing services. These elements included caregivers not recognizing their child’s behavior as problematic, the belief that the child would recover, and parental attitude/misconceptions (Al-Mohsin et al., 2020; Anand et al., 2018; Moody, 2017; O’Dell et al., 2022; Safavi et al., 2016). For example, common beliefs among caregivers of children screened at a pediatrics outpatient department of a hospital in India were that they thought the child was “just naughty” (reported by 84% of caregivers), that hyperactivity was a part of typical growth (64%) and/or that the child would improve with time (56%) (Anand et al., 2018). Similarly, in Safavi et al. (2016), respondents expressed the belief that the child would one day recover (55%) and that the child did not have any critical problems (41%). Some parents were avoidant of the possibility that their child may be eligible and meet the criteria of an ADHD diagnosis, dismissing problematic behavior as a result of reduced exercise (Moody, 2017). Other caregivers denied that their child had a problem even after a school screening intervention identified the child as at-risk of ADHD (Barry et al., 2016).

Of caregivers who acknowledged the ADHD symptoms/diagnosis in their child, there was a shared experience of not knowing where to go to access the right services. Caregivers reported a lack of knowledge about ADHD and the service delivery system (64.4%), not knowing where to go to seek help or whom to consult (30–75%) (Al-Mohsin et al., 2020; Safavi et al., 2016; Sayal et al., 2015; Yamauchi et al., 2015) and low awareness of treatment options (Anand et al., 2018). Caregivers often experienced feeling overwhelmed when navigating the health system alone (Spencer et al., 2021) with minimal guidance to find the right services (Paidipati et al., 2022). Not knowing where to go to receive support or access services presents a significant barrier to caregivers which may also contribute to the delay in help-seeking and receiving a diagnosis (Al-Mohsin et al., 2020; Yamauchi et al., 2015). A delay in accessing supportive care may contribute to ongoing unmet needs in children. For service non-users in a quantitative study, 32% of caregivers reported a lack of information about who could help, a belief that no one could help their child, and that specialists were not available for their child’s problem (Sayal et al., 2015).

From the clinician’s perspective, primary care clinicians have reported that parental misconceptions and false expectations of children’s behavior can make it challenging to engage parents in treatment utilization for ADHD (O’Dell et al., 2022). These barriers to diagnosis and treatment are driven by the beliefs of parents and the lack of understanding and awareness of the effects of ADHD on children.

##### Clinician Level of Expertise

Two studies identified a lack of understanding and expertise about ADHD among health providers as a negative influence on the process of diagnosis and treatment for children with ADHD (Honorio Neto et al., 2021; O’Dell et al., 2022). Primary care clinicians reported a lack of knowledge and confidence to effectively manage and provide comprehensive care for children with ADHD (O’Dell et al., 2022). While pediatricians were able to screen for ADHD, they often lacked the level of expertise necessary for diagnosing and treating children (Honorio Neto et al., 2021). Collaboration or integration between services would aid in the ease of obtaining diagnosis and treatment through sharing knowledge and information (Honorio Neto et al., 2021; O’Dell et al., 2022).

#### Stigma

In twelve studies, stigma was identified as an influential factor throughout the process of service access and utilization for ADHD diagnosis and treatment. Caregivers and primary care clinicians reported experiences of caregivers’ stigma associated with mental health issues and accessing mental health providers (Graves, 2017; Honorio Neto et al., 2021; O’Dell et al., 2022), and ADHD as a stigmatized diagnosis (Paidipati et al., 2022). Delays to first visiting an ADHD health provider can be partly explained by stigma (Anand et al., 2018). Caregivers reported concerns about speaking with a health provider (Spencer et al., 2021) and their family (Safavi et al., 2016) about their child’s ADHD symptoms due to fear, embarrassment or being judged (Graves, 2017; Safavi et al., 2016; Spencer et al., 2021). Furthermore, stigmatization of medication therapy resulted in caregiver hesitancy and resistance to initiating pharmacotherapy treatment due to social norms about medication overuse and labeling (Coletti et al., 2012; Graves, 2017; Paidipati et al., 2022). Stigma was also discussed as intersecting with ethnicity (Araujo et al., 2017; Moody, 2017; Paidipati et al., 2022), whereby caregivers experienced racial bias, which impacted their ability to access and utilize services to find the right treatments (Araujo et al., 2017; Saulsberry et al., 2020).

Within the educational setting, poor teacher insight into ADHD and harsh punishment for students with ADHD compared to students without ADHD resulted in greater stigmatization (Shippen et al., 2022). Stigmatization of ADHD in Black communities in the United States has led to some teachers refusing to acknowledge the possibility of ADHD symptoms in children due to what that label means for the child and community stigma (Moody, 2017). The issue of ethnic and racial bias is evident across two themes: stigma, and teacher awareness/acknowledgment, highlighting the overlap and related content of themes.

#### Importance of Parental Choice and Partnerships

Facilitating parental choice and partnerships with health providers in enabling access and ongoing utilization of services was reported in seven studies. Caregivers highlighted the importance of medical providers acknowledging that parents are the “expert” on their child’s health (Laugesen et al., 2017) and ultimately make the important decisions regarding care for their child (Coletti et al., 2012; Lu et al., 2022). Caregivers emphasized their role in advocating for their child’s treatment (Coletti et al., 2012; Spencer et al., 2021) and in deciding which treatments to pursue among a variety of treatments that would suit their child’s needs most (Lu et al., 2022). Partnerships with providers who practiced holistic approaches, collaborative and person-centered care were valued by caregivers and allowed the development of long-term relationships to form, enabling ongoing service utilization (Coletti et al., 2012; Paidipati et al., 2022; Spencer et al., 2021). Health providers having familiarity, understanding of the family’s circumstances, and working closely with the family enabled successful outcomes in care plans and ongoing utilization of services (Paidipati et al., 2022; Rezel-Potts et al., 2021; Saulsberry et al., 2020).

#### Education Services as an Integral Component

The school setting plays an active role in identifying behavioral and developmental issues in children and informs caregivers of the concerns regarding the child (Barry et al., 2016; Graves, 2017; Honorio Neto et al., 2021). Educational services also aid health providers in the assessment and diagnosis of ADHD by providing essential information and treatment interventions (O’Dell et al., 2022; Ogundele et al., 2022). Caregivers often receive the first referral to a specialist from school services or a recommendation to contact their physician after being identified as at-risk of ADHD (Anand et al., 2018; Barry et al., 2016; Sayal et al., 2015). Educational settings provide a wide range of treatment and support to caregivers and their children with ADHD, by offering guidance and information to accessing treatment (Paidipati et al., 2022), individualized education plans, mental health providers, and social workers (Saulsberry et al., 2020; Shippen et al., 2022). Collaboration between health providers and school settings contributes to improved treatment outcomes for children with ADHD and facilitates coordination and ease of care (O’Dell et al., 2022).

#### Referrals, Waiting Time, and Logistics

##### Obtaining a Referral and Extended Waiting Time

Extended waiting periods to obtain a referral or clinical assessment was a common barrier among caregivers when trying to access services for their child (Al-Mohsin et al., 2020; Flood et al., 2019; Fridman et al., 2017; Paidipati et al., 2022; Sayal et al., 2015). For example, caregivers across ten European countries reported a great deal or much difficulty in obtaining a formal ADHD diagnosis, and from 3 months up to 1.5 years waiting time to access a diagnosis as a result of variation in local diagnostic pathways and service models (Fridman et al., 2017). Caregivers have experienced difficulty in obtaining a referral or access to treatment from specific child and adolescent mental health services due to dismissal that the child’s condition is not severe enough (Rezel-Potts et al., 2021), poor availability of service resources (Flood et al., 2019; Zima et al., 2013) and lack of appropriate health professionals (Paidipati et al., 2022). The source of referral provided to caregivers may determine the delay to present to a health professional, as a referral from a teacher (1.8 years) led to caregivers presenting earlier, compared to a referral from a health practitioner (2.2 years) (Anand et al., 2018).

##### Logistics

More than two-thirds of all the included studies (*n* = 21) reported logistical barriers to service access and utilization, such as cost and financial difficulty (Barry et al., 2016; Benevides et al., 2017; Lu et al., 2022; Safavi et al., 2016; Sayal et al., 2015; Spencer et al., 2021; Zima et al., 2013), transportation issues (Paidipati et al., 2022; Spencer et al., 2021; Zima et al., 2013), lack of available time, inflexible caregiver work schedule, and child school/activity schedule (Barry et al., 2016; Hooven et al., 2018; Rezel-Potts et al., 2021; Yamauchi et al., 2015; Zima et al., 2013). Additionally, comorbidities such as oppositional defiant disorder increased psychiatric service utilization in children (Laugesen et al., 2020) and increased barriers to engagement in behavioral therapy interventions (Johansson et al., 2023). Moreover, different types of insurance, maternal education level, family income, family composition, and geographic distance to services were influential factors in caregiver’s practices when presenting to, accessing and utilizing diagnostic and treatment services (Laugesen et al., 2020; Lien et al., 2015; Pastor et al., 2017; Yamauchi et al., 2015). Publicly insured children in the United States were more likely to have a psychiatrist or psychologist as the first prescriber of medication, have contact with general practitioners and mental health providers, and be more likely to receive special education services than privately insured and uninsured children (Pastor et al., 2017). Initial diagnosis and treatment provider was associated with the type and utilization of services; those who received an initial diagnosis from a psychiatrist were more likely to commence combined, multimodal treatments (Lien et al., 2015), and caregivers who accepted medication treatment were less likely to engage in seeking multiple medical care providers (Tzang et al., 2014).

## Discussion

### Key Findings

This systematic review provides an update and extends the synthesis of the literature on the barriers and enablers to service access and utilization for children and adolescents with a diagnosis or symptoms of ADHD. The synthesis of the literature in this review brought out several major findings associated with ADHD knowledge levels among caregivers, clinicians, and teachers, challenges navigating the health system, financial and logistical barriers, as well as enablers such as partnerships and the educational setting.

The findings from this review are consistent with previous findings from systematic reviews related to the barriers and enablers of ADHD management and care (French et al., 2019; Wright et al., 2015). Our review extended the previous reviews by including both children with a diagnosis, undiagnosed children presenting with symptoms of ADHD and all care settings. Consistent with the main findings from an earlier review French et al. (2019), our review showed that a lack of awareness, poor acknowledgment and understanding of ADHD, and stigma as the most common barriers to ADHD service access and utilization by primary care clinicians, teachers, and caregivers. Lack of knowledge and awareness about ADHD results in poor acknowledgment of symptomatic children and increased stigma, directly preventing eligible children from accessing much-needed services (Moldavsky & Sayal, 2013; Tatlow-Golden et al., 2016). As noted in a previous review (French et al., 2019), there is an ongoing need for increased education and resources for primary care clinicians to facilitate access to diagnosis and care for individuals with ADHD.

Despite the findings being synthesized from various countries with different healthcare systems, our current review demonstrates that the common barrier of poor acknowledgment and awareness of ADHD has remained a significant challenge that still pervades clinical and educational settings globally. This finding demonstrates that strategies and interventions to increase family access and use of ADHD services may not be sufficient or effective in improving service provision, despite similar recommendations highlighted in previous reviews (French et al., 2019; Wright et al., 2015). The results from our review echo study findings from more than a decade ago, where poor awareness and attitudes toward ADHD were reported, suggesting the need for more effective education and awareness of ADHD and equitable access to services in a timely manner (Moldavsky & Sayal, 2013). Poor acknowledgment and awareness of ADHD have also been identified as a significant barrier to service access from the perspectives of various experts across the public and private health sectors regarding the failures of ADHD service provision in the United Kingdom (Young et al., 2021).

The stigma associated with an ADHD diagnosis, medication treatment, and mental health service utilization contributes to the ongoing challenges experienced by caregivers and their children across various cultures when help-seeking and utilizing specialist services (Anand et al., 2018; Moody, 2017; O’Dell et al., 2022; Paidipati et al., 2022; Safavi et al., 2016; Spencer et al., 2021). While our findings primarily reported on the experience of stigma from the perspective and experiences of caregivers, the barrier of stigma on service access is frequently reported in other studies in the context of general misconceptions and perspectives among clinicians (French et al., 2019; Young et al., 2021). Previous research emphasized that education and training for ADHD is needed to address the misconceptions and stigma of ADHD among caregivers, clinicians, and teachers (French et al., 2019; Moldavsky & Sayal, 2013; Tatlow-Golden et al., 2016; Young et al., 2021), including cultural safety training among clinicians and educators to improve practices (Paidipati et al., 2022; Shi et al., 2021). There is a need for clinicians to have an understanding of community stigma and cultural differences of ADHD as a barrier to help-seeking and service utilization (Wright et al., 2015).

Our findings about the challenges of navigating the health and service delivery system (Paidipati et al., 2022; Sayal et al., 2015; Spencer et al., 2021) are consistent with the broader literature on unmet needs associated with ADHD (Sikirica et al., 2015), indicating that not knowing which health providers to consult leads to a delay in seeking care for children, acting as a barrier to service access.

The barrier of extended waiting time and difficulty in obtaining a referral have been reported in previous research (Bonati et al., 2019; Sikirica et al., 2015; Tettenborn et al., 2008; Young et al., 2021). Waiting times may be influenced by the complexity of health system characteristics, including diagnostic pathways (Fridman et al., 2017), distribution of health care centers (Bonati et al., 2019), and the availability of health providers and resources for ADHD in health centers (Tettenborn et al., 2008). Previous research on care pathways highlights the importance of clinician adherence to guidelines, and thus ensuring effective and consistent implementation of diagnostic/care pathways could help address the barriers to accessing and utilizing services (Sayal et al., 2018; Stivala, 2021; Young et al., 2021). Our findings further emphasize the need for policy changes to service delivery and availability of ADHD specialists to enable timely access to services and prevent ongoing unmet needs, morbidity, and impairment due to long delays (Young et al., 2021).

We found that the developing partnerships between caregivers and healthcare providers is crucial throughout the process of diagnosis and treatment of ADHD in children to enable service utilization. Caregivers strongly valued clinicians who practiced holistic and person-centered care, enabling agency and choice of the caregivers, recognizing them as the decision maker for the management of their child’s ADHD, and supporting the navigation of the health service delivery system (Coletti et al., 2012; Laugesen et al., 2017; Lu et al., 2022; Spencer et al., 2021). This enabling factor has been reported in previous research (Davis et al., 2012). This demonstrates the importance in facilitating ongoing service utilization and supportive care outcomes through the development of long-term relationships between health providers and families managing ADHD.

From the perspective of clinicians, teachers, and caregivers, we found that the educational setting played an active and important role in the identification of children with ADHD symptoms and enabled access to diagnosis and supportive care. Educational settings are often the first identifier of ADHD symptoms in children and often provide the first referral or recommendation for the child to see a clinician or specialist (Anand et al., 2018; Barry et al., 2016; Sayal et al., 2015). The role of the school was also identified as an enabler of obtaining a referral, service access, school-based interventions, and care coordination, aligning with findings from previous literature (French et al., 2019; Young et al., 2021). Conversely, the educational system may create challenges or act as a barrier through poor knowledge among teachers and a lack of resources to provide adequate support and individualized education plans to children with ADHD (Paidipati et al., 2022). In a previous systematic review, primary care clinicians reported challenges with communication and coordinating care with educational providers (French et al., 2019). However, when teachers’ knowledge of ADHD and coordination of care are of an adequate level, the educational setting can provide invaluable services for children with ADHD and enable other service access and utilization (Anand et al., 2018; Ogundele et al., 2022; Paidipati et al., 2022; Zysset et al., 2023).

### Implications for Policy and Practice

The current review highlights a need for training and education of clinicians, health professionals and teachers to improve the recognition of and provision of care for children and adolescents with ADHD. Availability of educational resources for providers and caregivers would aid in increasing knowledge and awareness of ADHD, allowing for early identification of behavioral concerns and symptomatic children (Tatlow-Golden et al., 2016). It is important to also consider the educational system to provide supportive care and access to services when allocating resources and funding for ADHD interventions. Teachers and education providers need to be adequately trained to understand and manage ADHD to make use of the role that the school system plays in providing ADHD services for children. Service delivery systems should consider effective strategies to raise awareness of ADHD and to broaden their communications to ensure that communities are informed about the services that are available to them, particularly around specialist services for ADHD, and in languages and formats that are accessible to a range of cultures and communities. Policy changes to national service delivery systems should consider strategies to increase the physical and financial availability of health providers equipped to diagnose and treat ADHD in children to ultimately decrease barriers of waiting times and cost to service access and use.

### Recommendations for Future Research

Findings from the current review highlight a need to develop comprehensive ADHD education/training resources for caregivers, teachers, and clinicians to raise awareness of ADHD, its long-term impacts, and available services. Future research needs to identify and evaluate effective and cost-effective strategies to address the identified barriers of ADHD knowledge gaps, awareness, stigma, and physical/financial availability of services among caregivers, teachers and clinicians. Furthermore, developing an effective ADHD care pathway could aid caregivers in navigating health systems and finding the right services to reduce help-seeking delays and unmet medical needs.

### Strengths and Limitations

A strength of this systematic review is the comprehensive literature search strategy and use of multiple search terms. The inclusion of qualitative, quantitative, and mixed methodology studies strengthened this review by enabling a greater variety of literature, including rich descriptive data about the experiences of caregivers when accessing and utilizing services. Limitations of this review have been identified. Excluding non-English articles and grey literature possibly led to missing relevant articles and reports, which could have influenced the findings of this review. This review may have limited generalizability as the study focused on children and adolescents with ADHD and therefore, may not apply more generally to children with comorbidities, other similar conditions, and adults with ADHD.

## Conclusion

This systematic review updated and extended the literature on the barriers and enablers of service access and utilization for children and adolescents with ADHD diagnosis/symptoms through the perspective of caregivers, clinicians, and teachers. Barriers were identified, including acknowledgment and expertise of ADHD, navigating the health system, and difficulty obtaining a referral to services. Enablers included the development of partnerships between caregivers and clinicians and the importance of the educational sector in providing services and guidance.

These findings highlight the need for increased awareness of ADHD and its services as well as ongoing training and education of health and education professionals to improve the recognition of and provision of care for children and adolescents with ADHD. Policy changes and strategies for national service delivery systems to increase the physical and financial availability of ADHD specialists should be implemented to reduce time and cost barriers to service access. Future research should consider identifying and evaluating effective and cost-effective strategies to address these identified barriers and to support identified enablers.

## Appendix A

**Table A1.** Search Terms Used for Each Database.

1	TI OR AB	pediatri* OR paediatri* OR infan* OR child* OR teen*OR youth OR adolescen* OR TI toddler OR AB toddler OR (“baby” OR “babies”) OR “young people”	Search with AND
2	TI OR AB	“ADHD” OR “attention deficit hyperactivity disorder” OR “attention deficit disorder” OR “attention deficit disorder with hyperactivity”	
3	TI OR AB	health* N3 provision OR “health practi*” OR “healthcare” OR “health care” OR “health services” OR “health management” OR diagnos* OR treat* N3 access* OR service* OR “health utili*” OR access N3 service* OR service* N3 utili* OR “school base*”	
4	TI OR AB	enabl* OR facilitat* OR aid OR promot* OR barrier* OR hinder* OR obstacle OR challeng* OR difficult* OR issue* OR boundar*	

**Table A2.** Subject Headings Used for Each Database.

Search terms	1	2	3	4
MEDLINE complete	(MH “Child”) OR (MH “Adolescent”) OR (MH “Child, Preschool”) OR (MH “Infant”)	(MH “Attention Deficit Disorder with Hyperactivity”)	(MH “Health Services”) OR (MH “Health Services Accessibility”) OR (MH “Delivery of Health Care”)	
CINAHL complete	(MH “Child”) OR (MH “Adolescence”) OR (MH “Child, Preschool”) OR (MH “Infant”)	(MH “Attention Deficit Hyperactivity Disorder”)	(MH “Health Services Accessibility”) OR (MH “Child Health Services”) OR (MH “Adolescent Health Services”)	
APA PsycINFO	(DE “Child Health”) OR (DE “Adolescent Health”)	(DE “Attention Deficit Disorder with Hyperactivity”)	(DE “Health Care Services”) OR (DE “Health Care Access”)	(DE “Treatment Barriers”)
ERIC	(DE “Children”) OR (DE “Adolescents”)	(DE “Attention Deficit Hyperactivity Disorder”)	DE (DE “Health Services”) OR (DE “Access to Health Care”)	
Global health	(DE “Children”) OR (DE “Adolescents”)	(DE “Attention Deficit Hyperactivity Disorder”)	(DE “Health Services”)	

## Appendix B

**Table B1.** Quality Assessment Using the Mixed Methods Appraisal Tool.

Author/s (year)	Qualitative					Quantitative										Mixed methods					Total	QR
						Non-randomized					Quantitative descriptive											
	1.1	1.2	1.3	1.4	1.5	3.1	3.2	3.3	3.4	3.5	4.1	4.2	4.3	4.4	4.5	5.1	5.2	5.3	5.4	5.5		
Al-Mohsin et al. (2020)											Y	Y	Y	Y	CT						4.5	H
Anand et al. (2018)											Y	Y	Y	CT	CT						4	H
Araujo et al. (2017)	Y	Y	Y	Y	Y																5	H
Barry et al. (2016)											Y	Y	Y	CT	CT						4	H
Benevides et al. (2017)											Y	Y	Y	Y	Y						5	H
Coletti et al. (2012)	Y	Y	Y	Y	Y																5	H
Flood et al. (2019)	Y	Y	Y	Y	Y																5	H
Fridman et al. (2017)											Y	Y	Y	Y	Y						5	H
Graves (2017)	Y	Y	Y	Y	Y																5	H
Honorio Neto et al. (2021)																Y	CT	CT	Y	Y	4	H
Hooven et al. (2018)											Y	Y	Y	CT	Y						4.5	H
Johansson et al. (2023)											Y	Y	Y	CT	Y						4.5	H
Laugesen et al. (2017)	Y	Y	Y	Y	Y																5	H
Laugesen et al. (2020)																Y	Y	Y	Y	Y	5	H
Lien et al. (2015)											Y	Y	Y	CT	Y						4.5	H
Lu et al. (2022)	Y	Y	Y	Y	Y																5	H
Moody (2017)	Y	Y	Y	Y	Y																5	H
O’Dell et al. (2022)	Y	Y	Y	Y	Y																5	H
Ogundele et al. (2022)											Y	CT	Y	CT	Y						4	H
Paidipati et al. (2022)	Y	Y	Y	Y	Y																5	H
Pastor et al. (2017)											Y	Y	Y	Y	Y						5	H
Rezel-Potts et al. (2021)	Y	Y	Y	Y	Y																5	H
Safavi et al. (2016)											Y	Y	Y	CT	CT						4	H
Saulsberry et al., 2020)	Y	Y	Y	Y	Y																5	H
Sayal et al. (2015)						Y	Y	CT	Y	CT											4	H
Shippen et al. (2022)	Y	Y	Y	Y	Y																5	H
Spencer et al. (2021)	Y	Y	Y	Y	Y																5	H
Tzang et al. (2014)											Y	CT	Y	CT	Y						4	H
Yamauchi et al. (2015)											Y	N	Y	N	Y						3	M
Zima et al. (2013)						Y	Y	Y	Y	Y											5	H

*Note*. For each item: Yes (Y) = 1, No (N) = 0, Cannot tell (CT) = 0.5. Quality rating (QR): Low (L) = 1–2, Medium (M) = 3, High (H) = 4–5. CT has been defined as somewhat described, unclear or a vague explanation.

1. Qualitative 1.1. Is the qualitative approach appropriate to answer the research question? 1.2. Are the qualitative data collection methods adequate to address the research question? 1.3. Are the findings adequately derived from the data? 1.4. Is the interpretation of results sufficiently substantiated by data? 1.5. Is there coherence between qualitative data sources, collection, analysis, and interpretation?

3. Quantitative nonrandomized 3.1. Are the participants representative of the target population? 3.2. Are measurements appropriate regarding both the outcome and intervention (or exposure)? 3.3. Are there complete outcome data? 3.4. Are the confounders accounted for in the design and analysis? 3.5. During the study period, is the intervention administered (or exposure occurred) as intended?

4. Quantitative descriptive 4.1. Is the sampling strategy relevant to address the research question? 4.2. Is the sample representative of the target population? 4.3. Are the measurements appropriate? 4.4. Is the risk of nonresponse bias low? 4.5. Is the statistical analysis appropriate to answer the research question?

5. Mixed methods 5.1. Is there an adequate rationale for using a mixed methods design to address the research question? 5.2. Are the different components of the study effectively integrated to answer the research question? 5.3. Are the outputs of the integration of qualitative and quantitative components adequately interpreted? 5.4. Are divergences and inconsistencies between quantitative and qualitative results adequately addressed? 5.5. Do the different components of the study adhere to the quality criteria of each tradition of the methods involved?
